# Self-Supervised Visual Tracking via Image Synthesis and Domain Adversarial Learning

**DOI:** 10.3390/s25154621

**Published:** 2025-07-25

**Authors:** Gu Geng, Sida Zhou, Jianing Tang, Xinming Zhang, Qiao Liu, Di Yuan

**Affiliations:** 1 Guangzhou Institute of Technology, Xidian University, Guangzhou 510555, China; genggu_hbu@163.com; 2 School of Electrical and Information Engineering, Yunnan Minzu University, Kunming 650504, China; 3 School of Science, Harbin Institute of Technology, Shenzhen 518055, China; 4 National Center for Applied Mathematics, Chongqing Normal University, Chongqing 401331, China

**Keywords:** object tracking, self-supervised, image synthesis, domain adversarial learning

## Abstract

With the widespread use of sensors in applications such as autonomous driving and intelligent security, stable and efficient target tracking from diverse sensor data has become increasingly important. Self-supervised visual tracking has attracted increasing attention due to its potential to eliminate reliance on costly manual annotations; however, existing methods often train on incomplete object representations, resulting in inaccurate localization during inference. In addition, current methods typically struggle when applied to deep networks. To address these limitations, we propose a novel self-supervised tracking framework based on image synthesis and domain adversarial learning. We first construct a large-scale database of real-world target objects, then synthesize training video pairs by randomly inserting these targets into background frames while applying geometric and appearance transformations to simulate realistic variations. To reduce domain shift introduced by synthetic content, we incorporate a domain classification branch after feature extraction and adopt domain adversarial training to encourage feature alignment between real and synthetic domains. Experimental results on five standard tracking benchmarks demonstrate that our method significantly enhances tracking accuracy compared to existing self-supervised approaches without introducing any additional labeling cost. The proposed framework not only ensures complete target coverage during training but also shows strong scalability to deeper network architectures, offering a practical and effective solution for real-world tracking applications.

## 1. Introduction

Visual object tracking is an essential issue in the field of computer vision involving the task of estimating the state of the target in subsequent frames based on a given target object in the initial frame. The issue of achieving stable and efficient target tracking from these diverse sensor data has become a research hotspot in recent years with the wide application of various sensors such as cameras, infrared sensors, etc., in fields such as autonomous driving, intelligent security, and augmented reality. The proven success of transformer architectures [1] across multiple computer vision domains has made them a popular choice for the design of visual tracking algorithms [2,3,4]. Despite their success, transformer-based tracking methods necessitate a substantial volume of video data for effective training. Due to the expense and time-consuming nature of manually annotating data, it is crucial to investigate self-supervised visual target tracking as a viable alternative. Given the current widespread use of deep neural network models, there are two key issues that self-supervised learning needs to address: first, designing a pretext task that enables the model to learn discriminative feature representation of the tracked target; and second, finding more effective ways to train deep neural networks. Researchers have conducted extensive exploration to address both of these issues.

In the design of pretext tasks, existing self-supervised methods such as UDT [5], Self-SDCT [6], and CycleSiam [7] perform bidirectional tracking by randomly cropping the template region in the initial frame, then calculating the consistency loss of the targets in the initial frame before and after tracking as the supervision signal. On the other hand, the S2SiamFC tracker [8] generates training pairs by randomly cropping the template region in the image and pairing it with the original image. This approach utilizes an adversarial masking strategy to blur the appearance of the target, enabling the model to learn contextual information related to the target. Additionally, the USOT tracker [9] employs unsupervised optical flow and dynamic programming methods to crop moving objects instead of random cropping while adding a cycle memory learning scheme to enable the model can be trained on a longer time span. Despite the aforementioned methods using different cropping strategies in the initial frame, they can still fail to completely enclose the entire target and often capture only a part of it. Consequently, the model is unable to learn complete information related to the target, which leads to the complete target not being included during actual testing. Moreover, these models are highly susceptible to interference from similar objects, resulting in poor robustness.

As with other self-supervised methods such as MAST [10], differentiable memory modules have been proposed to enhance self-supervised tracking algorithms. Approaches such as JLSTC [11], ContrastCorr [12], and others [13,14,15,16] explore the leveraging of spatiotemporal contextual information in videos for self-supervised representation learning. In addition, Li et al. [17] synthesized training data by applying transformations on given labeled targets, thereby enhancing the robustness of the tracker. These approaches do not directly conduct end-to-end training on unlabeled data, and achieve commendable performance when applied to trackers; however, they are only suitable for training shallow networks. When applied to deep networks such as DiMP [18] and ToMP [2], their performance often significantly lags behind supervised learning methods.

**Figure 1:**
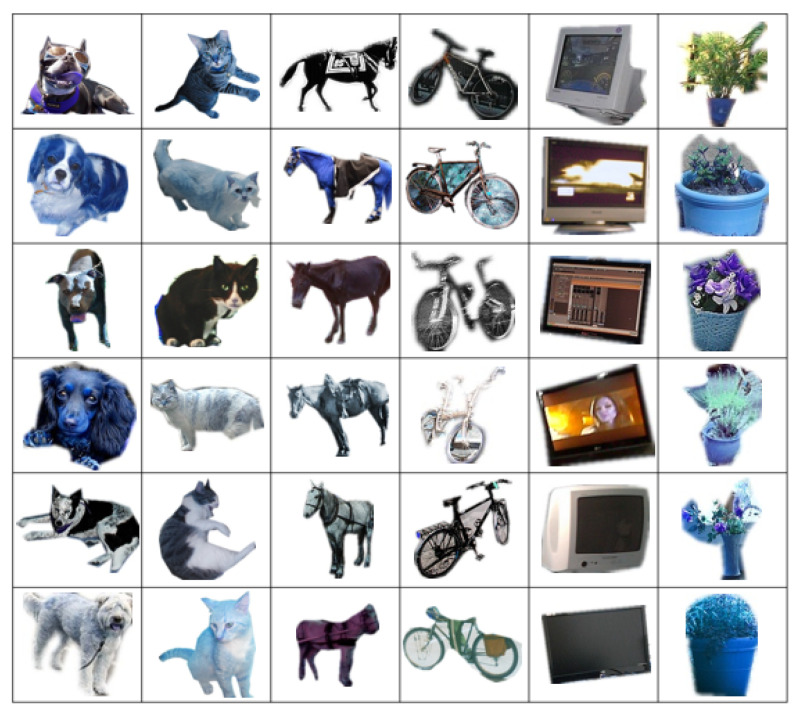
Overview of the target object database. The figure shows a random selection of target objects from the target object database, with the objects in each column belonging to the same category. This database is used to synthesize realistic training pairs in our self-supervised tracking framework.

Existing self-supervised methods often rely on incomplete object representations during training, resulting in inaccurate localization during inference. Moreover, their effectiveness significantly degrades when applied to deep neural networks. In this work, we propose a self-supervised visual tracking method via image synthesis and domain adversarial learning to solve the issues mentioned above. First, to tackle the problem of current methods being unable to crop a complete target in the initial frame, we introduce a real-world target and then synthesize it into both the initial and subsequent frames using image synthesis techniques. In this way, we establish an extensive target object database that can ensure the quality and quantity of synthesized targets. Examples of specific objects in the database are presented in Figure 1. Second, our proposed method is fully capable of training deep neural networks. By synthesizing targets in video frames, we can obtain complete and accurate target bounding boxes. This allow the model to be trained in a supervised manner, which can be applied to the training of deep networks. Third, in addition to directly using synthesized data for training, we employ domain adversarial training to reduce the difference between synthesized and real data. Such differences include obvious edges of synthesized targets and irregular color transitions between synthesized targets and original images. These differences may lead to the model overfitting on the training data, as learning these differences can cause the model to converge quickly without actually learning any target-related features. Through domain adversarial learning, the model can learn truly discriminative features related to the target. Finally, we fine-tune the tracker with a small amount of labeled data to further improve its tracking performance on downstream tasks.

Extensive experiments demonstrate that our self-supervised tracker achieves competitive tracking performance with supervised trackers without increasing the cost of data annotation. The main contributions of this work are summarized as follows:

- We propose a novel self-supervised tracking framework that leverages image synthesis and domain adversarial learning to enable effective training of deep tracking networks by using complete target instances without manual annotations.
- We incorporate domain adversarial learning into the training pipeline to reduce the domain gap between synthetic and real data, which mitigates overfitting and facilitates the extraction of robust and discriminative features.
- Extensive experiments on multiple benchmarks demonstrate that our method achieves comparable performance to supervised methods.

## 2. Related Work

### 2.1. Visual Tracking Method

In recent years, single-stage visual trackers have become mainstream in the field of visual object tracking [19,20,21]. These models utilize a unified encoder to simultaneously perform feature extraction and feature interaction, simplifying the overall architecture and improving efficiency. OSTrack [22] was the first to successfully adopt this design, demonstrating significant gains in both tracking accuracy and speed. Building upon this foundation, subsequent works [23,24,25] have proposed various enhancements to the attention mechanism to further refine the interaction between the template and search regions. Despite their strong performance, these one-stage trackers often struggle with long-term tracking scenarios, particularly when the target undergoes significant appearance changes or occlusions. To address this limitation, several recent approaches [26,27,28] have introduced multi-template mechanisms, enabling the model to maintain memory of the target over time. For instance, ODTrack [28] learns the joint dependencies between multiple templates and the current search region within the encoder, while MambaLCT [29] compresses historical target representations into a compact set of feature tokens, which are then updated using the Mamba architecture, improving long-term reasoning capability. Similarly, LMTrack [30] retrieves template patches from previous frames and performs joint modeling with the current search region to enhance robustness in challenging conditions.

While these advanced trackers achieve state-of-the-art performance, their success relies heavily on large-scale annotated datasets. With the increasing complexity and parameter count of modern tracking models, the demand for labeled data has grown significantly, posing a barrier to scalability. In this paper, we propose a self-supervised training framework that leverages synthetic images to replace manually annotated data. This approach greatly reduces the dependency on human-labeled data, providing an efficient and scalable solution for training deep tracking models without compromising performance.

### 2.2. Self-Supervised Visual Tracking

In recent years, trackers [2,31,32] have become increasingly reliant on large-scale datasets for training (e.g., ODTrack [28], SWinTrack [33]), highlighting the advantages of self-supervised learning that eliminates the need for labeled data. Among existing self-supervised tracking approaches, several different methods [5,7,9,34] have adopted a strategy that randomly crops the template region in the initial frame as the target, then enforces consistency by computing a loss between the target appearance before and after bidirectional tracking. Other methods [11,12,34] exploit the spatiotemporal context across video frames; for instance by colorizing objects in future frames utilizing information extracted from previous frames. Although these methods have achieved some success, they fail to capture the complete target during training; as a result, the tracker often focuses on only a part of the target during inference, leading to degraded tracking performance.

Recently, several trackers such as SimTrack [35], STTrack [36], and MixFormer [37] have pioneered the use of transformer-based architectures and achieved remarkable performance. For this type of tracker, MAT [38] applies random masking to both the template and search regions and enforces mutual reconstruction based on their cross-region correlations. Diff-Tracker [39] leverages the rich knowledge embedded in pretrained diffusion models to perform unsupervised visual tracking, including their understanding of image semantics and structural information. The method proposed in [17] generates labeled data for training trackers by employing a crop–transform–paste strategy. ARPTrack [40] is conditioned on the target’s historical appearance to generate future video frames using a diffusion model, enabling unsupervised pretraining of the tracker on these synthesized sequences. Furthermore, methods have utilized the SAM model [41] to help reduce the use of labeled data thanks to its powerful performance and zero-shot generalization ability. For instance, SAM2MOT [42] directly applies the pretrained SAM to the multi-object tracking task without any fine-tuning while maintaining high performance and significantly reducing the demand for labeled data. The method proposed in [43] uses the SAM model to generate pseudolabels, helping to to lower the cost of labeled data.

Unlike the aforementioned methods, in this study we propose constructing a target object database. We then randomly select target objects from this database and combine them into video frames to obtain accurate target bounding boxes, enabling the use of complete targets to train the model in a supervised manner.

### 2.3. Image Synthesis Method

With the rapid development of deep learning, the demand for data in model training is increasing. However, acquiring labeled data is expensive and time-consuming. Constrained by the scarcity of training data, many researchers resort to image synthesis methods to augment training data [44,45,46]. Zhang et al. [44] utilized image synthesis to generate thermal infrared images, facilitating end-to-end training of thermal infrared trackers and alleviating the scarcity of thermal infrared image data. Li et al. [17] enhanced and transformed the targets in the first frame of each video sequence, synthesizing them into subsequent frames to enhance the tracker’s robustness against interference from similar objects. Kerim et al. [47] employed image synthesis to generate a person-tracking dataset under various adverse weather conditions, addressing the insufficient pedestrian data in harsh weather conditions. Khoreva et al. [48] synthesized plausible future video frames using annotations provided on the first frame of each video, enabling model training with only a small amount of labeled data. Song et al. [49] proposed a self-supervised object synthesis framework that can comprehensively solve the object synthesis task within a unified model while changing the perspective, geometric shape, color, and shadow of the generated objects without the need for manual annotation; Chen et al. [50] utilized artificial intelligence to synthesize data to address the problem of fault data in the railway system being difficult to access. These methods all directly utilize synthesized data or mix synthesized data with real data to train models, but ignore the differences between synthesized and real data.

In this work, we incorporate domain adversarial training into the regular training process to prevent models from quickly overfitting on synthesized data and failing to learn genuine discriminative features, which can help the model to effectively extract the most discriminative features.

### 2.4. Domain Adversarial Learning

Domain adversarial learning aims to reduce the distributional discrepancy between the source and target domains by learning domain-invariant representations. A representative approach in this field is the Domain Adversarial Neural Network [51], which introduces a gradient reversal layer to enable joint optimization of the feature extractor and domain classifier in an end-to-end manner. To improve training stability and generalization performance, the method proposed in [52] introduced Smooth Domain Adversarial Training, which regularizes the adversarial process and enhances the effectiveness of domain alignment. Zhou et al. [53] extended adversarial learning to the domain generalization setting by designing a learning objective that encourages the classifier to correctly predict labels while simultaneously deceiving the domain discriminator. In addition to conventional classification tasks, Uplavikar et al. [54] utilized adversarial learning to enhance underwater images by aligning visual characteristics across different aquatic environments, while Chen et al. [55] combined self-training with domain adversarial learning to address the limitation that traditional methods align feature distributions without ensuring their discriminative capacity in the target domain. From a theoretical perspective, recent work by [56] has introduced a novel generalization bound for domain adaptation based on the f-divergence. This development unifies and extends existing theoretical results, including those of DANNs. In the context of reinforcement learning, domain adversarial optimization has been employed to improve the robustness of learned representations against visual domain shifts, enabling zero-shot generalization to unseen environments [57]. In industrial settings, the Domain Adversarial Graph Convolutional Network [58] jointly models class labels, domain labels, and the structural relationships among data samples to tackle mechanical fault diagnosis under varying working conditions.

However, despite such a wide range of applications, domain adversarial learning has rarely been explored in tracking. In this work, we utilize domain adversarial learning to mitigate the differences between synthesized and real data in the process of self-supervised training. We hope that this can provide some inspiration for future exploration.

## 3. Methods

**Figure 2:**
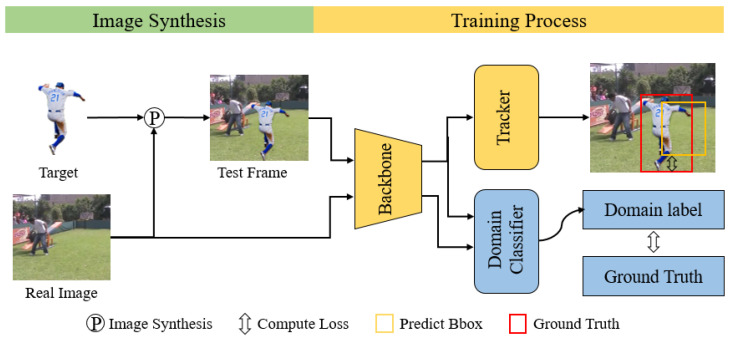
Overview of the proposed self-supervised framework. In the image synthesis part, our method randomly selects target objects from the target object database and synthesizes them into video frames to form training pairs. During the synthesis of test frames, we apply image transformations to the target objects. During the training process, we add a domain classifier (blue) behind the backbone of the tracker, which is used for domain adversarial training. Its primary function is to perform domain classification on the images inputted to the network. The other part (yellow) represents the tracker used for training.

We propose a self-supervised training method for visual tracking based on image synthesis and domain adversarial training. Figure 2 illustrates the training process. Our method begins by constructing a target object database, which is used to synthesize training data with unlabeled video sequences. The construction process is described in detail in Section 3.1. Next, Section 3.2 elaborates on how we synthesize training pairs by embedding target objects from the database into video frames. These synthetic pairs allow us to perform self-supervised training using complete target objects, addressing the limitation that existing methods typically rely on partial or randomly cropped targets. Finally, Section 3.3 presents our integration of domain adversarial learning into the training pipeline from Section 3.2. Incorporating domain adversarial training effectively mitigates the discrepancy between synthetic and real-world images, enhancing the generalization capability of the tracker.

### 3.1. Construction of the Target Object Database

The idea of synthesizing a target object into a video frame to train the object tracking model using the complete target object in the unlabeled data is a straightforward one. Building upon this thought and aiming to bring the synthesized data closer to the real data, we propose synthesizing real-world targets into video frames for tracking. To achieve this, we collect target segmentation labels from CoCo [59] and YouTube-VOS [60] datasets and filter out labels that do not meet the requirements, such as targets that are incomplete or too small. Subsequently, we establish a database of about a million different target objects. Figure 1 illustrates some of the target objects in the database.

As can be seen from Figure 1, our target objects are all real objects in natural scenes, meaning that they can be directly used by the tracker as the tracking target. Using this target database, it is possible to train the tracker on any original video sequence. Specifically, we can select targets from the target object database and synthesize them into the video sequence, from which we obtain the bounding boxes of the targets in the video sequence. In this way, we transform the unlabeled video sequence into the labeled video sequence without any extra cost, after which the tracker can be trained according to the normal procedure.

### 3.2. Image Synthesis Method

The process of our image synthesis method is illustrated in the image synthesis part of Figure 2. The main idea involves randomly selecting a target from the target object database and simultaneously extracting two frames from the video sequence. Then, the target and the transformed target are synthesized into the two frames as template frames and test frames, respectively. Finally, the template frame and the test frame are paired to form a training pair. The detailed generation steps for the template frame and the test frame are described below.

For the generation of the template frame $I_{temp}$, we randomly select a target *z* from the target object database *Z* and an original template frame $x_{t}$ from the video sequence $X=\{x_{i}\}$. The subscripts *i* and *t* denote the respective frame sequence numbers. Next, we synthesize the target *z* into the video frame $x_{t}$ in a pasted fashion to obtain the final template frame for training. The paste operation is denoted as P(·). In mathematical language, the above process can be described as follows:

(1) $$\begin{array}{cc} z=\; & S(Z), \end{array}$$

(2) $$\begin{array}{cc} x_{t}=\; & S(X), \end{array}$$

(3) $$\begin{array}{cc} I_{temp}=\; & P(x_{t},z,\left(x_{t},y_{t}\right)), \end{array}$$

where the function S(·) represents the random selection operation. For the image synthesis operation $P(x_{t},z,\left(x_{t},y_{t}\right))$, we first select a location $\left(x_{t},y_{t}\right)$ within the video frame for synthesizing. Then, the mask of the target object $mask_{z}$ is padded to match the size of the video frame based on the selected location. Finally, the synthetic image is generated as follows: $I_{temp}=mask_{z}\cdot z+\left(1-mask_{z}\right)\cdot x_{t}$, where the value of ${\mathrm{mask}}_{z}$ is 1 at the target position and 0 elsewhere.

**Figure 3:**
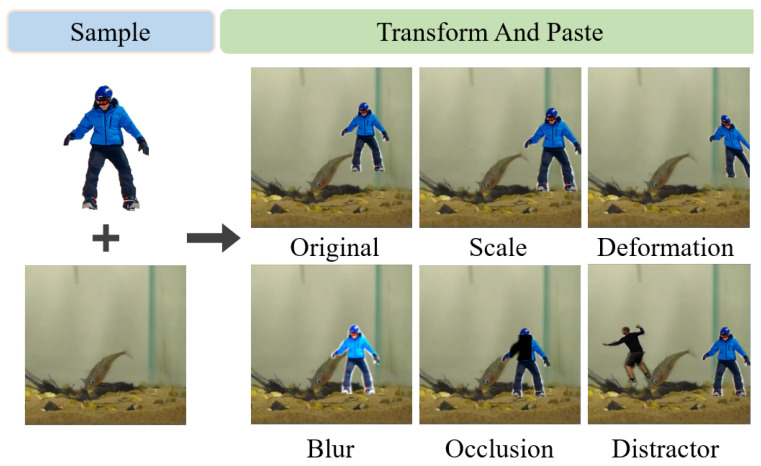
Main steps of image transformation operations. The left part is the object extracted from the target object database and the video frame extracted from the video, while the right side lists the five transformation operations and their effects.

The generation process of the test frames is similar to that of the template frames. First, we randomly extract the original test frame $x_{\left(t+n\right)}$ from the video sequence. To simulate the change of the target in the real world, we apply random transformation operations to targets before synthesizing them into the test frame. These operations include blurring, scaling, occlusion, deformation, and distractors (similar object interference). The synthesis process for the test frame can be represented as follows:

(4) $$\begin{array}{cc} z^{\prime }= & T_{j}\left(z\right), \end{array}$$

(5) $$\begin{array}{cc} x_{t+n}= & S(X), \end{array}$$

(6) $$\begin{array}{cc} I_{test}= & P(x_{t+n},z^{\prime },\left(x_{t+n},y_{t+n}\right)), \end{array}$$

where the symbol $z^{\prime }$ represents the target after applying the *j*-th transformation operation $T_{j}\left(z\right)$ and the letter *n* is an integer between 1 and 30 which ensures the sampling distance between the original template frame and the original test frame does not exceed 30 frames. An example of the image synthesis effect before and after target transformation is shown in Figure 3; for detailed parameter settings and strategies, please refer to the Experimental Details section.

Ultimately, our approach enables synthesizing target objects at designated locations in unlabeled video frames, thereby generating pseudo-labels and constructing training samples. This allows us to perform self-supervised training of the tracker using unlabeled video data. Unlike existing self-supervised methods that often rely on partial targets during training which leading to the model focusing on only a part region of the target during inference, our method leverages complete target objects for training and effectively avoiding this limitation and improving tracking accuracy.

### 3.3. Domain Adversarial Training

If the training process is conducted solely on synthesized data, the model will be prone to overfitting due to the tendency to capture differences between the synthesized target and the video frames, especially at the edges of the target. In this case, the model will perform very well during training but poorly during testing, as it cannot extract discriminative features related to the target. Specifically, on the test image, the model cannot capture the edges formed by the synthetic target, meaning that it has difficulty tracking the target; at the same time, because the discriminative features related to the target are not learned, the tracking is extremely susceptible to interference from similar targets. In this work, we address this issue by employing domain adversarial training, which is a technique derived from domain adaptation theory.

Concretely, we add a domain classifier behind the feature extractor (Encoder); the detailed method structure is demonstrated in the forward tracking part of Figure 2. The domain classifier consists of two fully connected layers, and primarily aims to distinguish whether the input images belong to synthesized or real images. In terms of input, the template branch and test branch input both real images and synthetic images; only synthetic images are used when training the tracker, while both synthetic and real images are used when training the domain classifier. The specific training process involves two alternating steps. In the first step, consisting of domain classifier training, the forward process calculates the domain classification loss of all input images using binary cross-entropy loss, and the tracker’s loss is ignored. In the process of updating the parameters, we freeze all parameters of the tracker and update the parameters of the domain classifier in the direction of reducing the domain classification loss. The second step consists of tracker training, in which the forward process simultaneously computes the tracking loss on synthesized images and the domain classification loss on all input images. During parameter updating, we freeze all parameters of the domain classifier and the parameters of the tracker are optimized in the direction of increasing the domain classification loss and decreasing the tracking loss. It is important to note that the calculation of the tracking loss depends on the loss calculation method of the original tracker.

To facilitate understanding, we use mathematical notation to restate the above process. We use the symbol $l_{t}\left(\cdot \right)$ to represent the total tracking loss of the tracker and the symbol $l_{c}\left(\cdot \right)$ to represent the classification loss of the domain classifier. Then, the domain classifier training loss can be represented as follows:

(7) $$\begin{array}{cc} u_{temp}=\; & M(x_{t},l_{temp}), \end{array}$$

(8) $$\begin{array}{cc} u_{test}=\; & M(x_{t+n},l_{test}), \end{array}$$

(9) $$\begin{array}{cc} L_{domain}=\; & l_{c}\left(u_{temp},y_{c}\right)+l_{c}\left(u_{test},y_{c}\right), \end{array}$$

where M(·) represents the image mixing operation and $y_{c}$ is the domain label indicating whether the image belongs to synthesized or real images. Similarly, the tracker training loss can be represented as follows:

(10) $$L_{tracker}=\alpha \cdot l_{t}\left(I_{test},y_{t}\right)-\beta \cdot L_{domain}$$

where $y_{t}$ represents the label of the test frame, while α and β are scalar weightings used to regulate the contribution ratio of each loss.

The main idea of the whole process is to force the tracker to avoid tracking by exploiting the difference between the synthetic target and the real target. When this difference is used for tracking, the domain classifier distinguishes whether the image is real or synthetic, meaning that the classification loss is reduced. However, because we optimize the domain classifier in the direction of increasing the classification loss during training, the tracker must extract discriminative features related to the object in order to track effectively. Ultimately, by integrating domain adversarial learning into the training process of the tracker, it is possible to prevent the tracker from overfitting on synthetic data, ensuring good generalization performance in real-world testing.

## 4. Experiments

In this section, we employ the ToMP tracker as the baseline and train it using our proposed method. The training details are outlined in Section 4.1. To validate the effectiveness of our proposed method, we conduct comparative experiments between our tracker and the original ToMP tracker in Section 4.2. Subsequently, in Section 4.3.1 we perform ablation experiments on various components of our method to demonstrate their indispensability. In Section 4.3.2, we visualize the features extracted by the model before and after adversarial training to show the effectiveness of the adversarial training process in our tracker. In Section 4.4, we verified the effectiveness and competitiveness of our tracker when trained with limited budget samples by comparing it to state-of-the-art trackers trained with extensive labeled training samples on five standard benchmark datasets (OTB100 [61], UAV123 [62], GOT10k [63], LaSOT [64], and TrackingNet [65]). Finally, in Section 4.5 we visualize the tracking results of our tracker to other representative trackers on the LaSOT dataset to demonstrate its effectiveness in complex tracking scenarios.

### 4.1. Experimental Details

We trained ToMP [2] on the data collected from five datasets, including LaSOT [64], GOT-10k [63], ImageNet VID [66], YouTube-VOS [60], and TrackingNet [65]. It is worth noting that the ground truth labels of these datasets are not available in our method. During training, we utilized AdamW on an NVIDIA GeForce RTX 3090 GPU. Additionally, to ensure a more natural color transition between the target and background in synthesized images, we preprocessed the target objects using a Gaussian kernel before compositing them, allowing the target objects to appear in the image linearly.

The training process consisted of 340 epochs, with each epoch comprising 30,000 images and a batch size set to 20. Throughout the entire process, the domain classifier and tracker were alternately trained. In each alternating cycle, the domain classifier was trained for two epochs and the tracker was trained for six epochs. During the training of the tracker, the parameters of the domain classifier were frozen and the hyperparameters in the loss function were set to α = 100 and β = 1. Additionally, ToMP has two sets of parameters trained with different learning rates; for the first four epochs, these were set to 2 $\times \;10^{-5}$ and 1 $\times \;10^{-5}$, respectively, then decreased to 4 $\times \;10^{-6}$ and 2 $\times \;10^{-6}$ after the fourth epoch. During training of the domain classifier, the parameters of the tracker were frozen and the learning rate was set to 1 $\times \;10^{-5}$ for the first epoch and decreased to 2 $\times \;10^{-6}$ for the second epoch. Finally, after the end of adversarial training, we fine-tuned the tracker on the labeled training dataset of the original tracker until convergence.

For the detailed parameter settings of the target transformations in the synthetic image generation process described in Section 3.2, we implemented the occlusion transformation using a Gaussian filter kernel with a size of 9. The scaling factor for scale transformation was randomly sampled from the range [0.7,1.3]. Affine transformations were used to simulate the deformation of the target. To introduce distractor variations, we additionally synthesized and placed two visually similar targets within the same region. Finally, we applied color augmentation to simulate additional blur effects. Specifically, the contrast was randomly adjusted within the range [0.9,1.1] and the brightness was randomly varied within the range [−10,10].

### 4.2. Contrast Experiment

**Figure 4:**
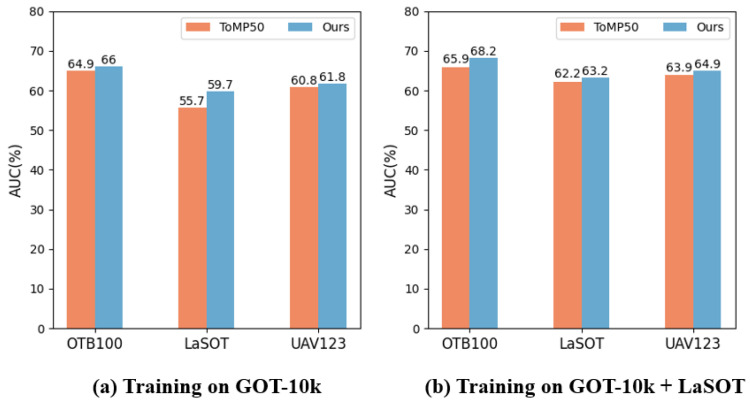
Performance comparison between the original ToMP and our tracker, using AUC scores as the performance metric. The left shows the comparison of tracker performance trained only on GOT-10k, while the right shows the comparison of tracker performance trained on both GOT-10k and LaSOT.

To verify that our method can improve the tracker’s performance without increasing data collection costs, we compared our tracker with the original ToMP [2]. Specifically, both our tracker and the original tracker were trained to converge on the training data, then evaluated on three benchmarks: OTB100 [61], LaSOT [64], and UAV123 [62]. We conducted two sets of comparative experiments based on the number of labeled datasets used during training: GOT-10k [63] and GOT-10k+LaSOT. The experimental results are shown in Figure 4.

The evaluation results when using only GOT-10k as the training dataset are shown in Figure 4a. It can be observed that the AUC scores of ToMP improved by 1% on OTB100, 4% on LaSOT, and 1% on UAV123 after training with our method. This indicates that our method can indeed improve the model’s performance without increasing data collection costs. To validate the effectiveness of our method with more annotated data, we conducted a second set of comparative experiments with increased training data. The experimental results are shown in Figure 4b. Similar to Figure 4a, our tracker showed improvements on all three benchmarks. This demonstrates that our method can enhance the tracker’s performance regardless of whether a small amount or larger amount of labeled data is used for training.

### 4.3. Ablation Study

#### 4.3.1. Effectiveness of Each Component

To validate the effectiveness and importance of each component in our method, we conducted ablation experiments on its two parts, namely, image transformation and domain adversarial training. Models were trained using unlabeled data from the GOT-10k, LaSOT, and TrackingNet datasets. After pretraining the models on unlabeled datasets using our method, the models were then trained on the labeled data until convergence. The detailed evaluation results are shown in Table 1.

As can be seen from Table 1, the worst performance is achieved when neither module is used. When using either module individually, the performance improves significantly. Comparing the second and fourth rows, it can be seen that the success rate curve (AUC) is improved by 2.3% and that the position prediction accuracy is improved by 1.9% after using the image transformation operations. This indicates that the image transformation operations help the tracker to learn the discriminative features of the target. Comparing the third row with the fourth row shows that the AUC is improved by 2.4% and that the location prediction accuracy is improved by 1.4% after using the domain adversarial training module. The larger improvement of the tracker before and after using domain adversarial training indicates that domain adversarial training is effective in mitigating the adverse effects of synthetic data. To illustrate the effect of domain adversarial more clearly, we visualize the features extracted by the model before and after domain adversarial training in Section 4.3.2.

#### 4.3.2. Feature Visualization of the Effectiveness of Domain Adversarial Training

**Figure 5:**
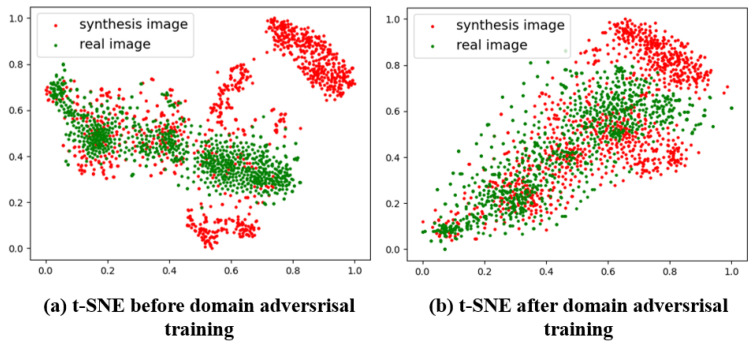
t-SNE feature clustering visualization graph: (**a**) the feature visualization graph before adversarial training and (**b**) the feature visualization graph after adversarial training. In each graph, red points represent features from synthetic images, while green points represent features from real images.

To provide a clearer demonstration of the impact of domain adversarial training on the model, we utilize the t-SNE clustering method [67] to visualize the features extracted by the model before and after adversarial training. Specifically, we mixed 1000 real images with 1000 synthetic images and fed them into the model, then extracted the features output by the encoder of the model and performed two-dimensional t-SNE clustering on them to obtain the clustering results. Finally, we normalized these two-dimensional clustering results for visualization. The detailed results are shown in Figure 5.

Comparing the two images in Figure 5 clearly shows that the features of the real images and synthetic images can be distinctly separated prior to adversarial training. This indicates that the model extracts significantly different features from the synthetic and real images. In other words, the model overfits to the synthetic dataset, and its performance on synthetic images does not accurately represent its performance on real images; consequently, a model trained on synthetic images will perform poorly on real images. On the other hand, a significant reduction in the differences between the features extracted from the two types of images can be observed after adversarial training. This suggests that the model extracts features shared by both synthetic and real images for tracking, meaning that its performance on synthetic images is now indicative of its performance on real images. From the feature visualization, it can be concluded that adversarial training effectively mitigates the overfitting phenomenon observed in the model trained on synthetic data.

### 4.4. State-of-the-Art Comparison

Next, we fine-tuned our tracker on labeled data to convergence. In this section, we present comparisons of our tracker with some state-of-the-art trackers on five challenging benchmark datasets to verify the effectiveness of our proposed approach. The benchmark datasets are GOT-10k [63], TrackingNet [65], OTB100 [61], LaSOT [64], and UAV123 [62].

**GOT-10k** [63]. To demonstrate the superior performance of our tracker, we evaluated it on the GOT-10k dataset and compared it with other trackers. The GOT-10K test set consists of 180 video sequences, and the test method is similar to the test method on the TrackingNet data. We uploaded tracking results of our tracker on the GOT-10k test set to the official website for evaluation and compared our results with those of several state-of-the-art trackers. The evaluation metrics used in GOT-10K include the Average Overlap (AO), Success Rate at threshold 0.5 (SR0.5), and Success Rate at threshold 0.75 (SR0.75). The compared state-of-the-art trackers include BANDT [68], SiamTPN [69], SiamGAT [70], SiamR-CNN [71], PrDiMP-50 [72], Ocean [73], SiamFC++ [74], DiMP-50 [18], SiamRPN++ [75], and STMTrack [76]. Detailed performance comparison results are provided in Table 2.

From Table 2, it is evident that our tracker achieves the highest scores in AO and SR0.5 among the various trackers, ranking second in SR0.75. Specifically, our tracker outperforms the second-ranked SiamR-CNN by 1.5% in AO and surpasses the third-ranked BANDT by 1.9%. In terms of SR0.5, our tracker exhibits a notable performance advantage of 3.6% over the second-ranked tracker. Lastly, in terms of SR0.75, our tracker trails the top-performing tracker by only 0.5%. This analysis demonstrates that our tracker holds a significant competitive edge among these state-of-the-art trackers, highlighting its superior performance across multiple evaluation metrics.

**TrackingNet** [65]. We also evaluated our tracker on the large-scale TrackingNet dataset, which consists of 511 video sequences without publicly available ground truth labels. Specifically, we tested our tracker on the test split of TrackingNet, then submitted the tracking results to the official website for evaluation. Subsequently, we compared our tracker with other state-of-the-art trackers, including BANDT [68], SiamTPN [69], TrTr [3], TrDiMP [79], HiFT [80], KeepTrack [81], PDiMP-50 [72], Ocean [73], SiamFC++ [74], DiMP-50 [18], SiamRPN++ [75], and SiamDW [77]. The comparison is based on metrics such as Precision, Normalized Precision, and AUC scores. Detailed results are presented in Table 3.

From Table 3, it can be observed that our tracker achieves the best performance across all three of the Precision, Normalized Precision, and AUC score metrics. Compared to the second-ranked tracker, BANDT, our tracker outperforms it by 0.4%, 0.8%, and 0.2%, respectively, in these three metrics; furthermore, our tracker maintains a significant lead over the third-ranked tracker, demonstrating that the tracker trained using our method can achieve competitive tracking performance compared to state-of-the-art trackers.

To demonstrate the effectiveness of our approach, we compared a version of our tracker without fine-tuning to the other self-supervised methods. The comparison results are shown in the starred results in the table. From these results, it can be seen that our method demonstrates a significantly higher performance advantage compared to existing methods, further proving the superiority of our approach.

**OTB100 [61]**. We evaluated our tracker on the classic benchmark dataset OTB100, which consists of 100 annotated short video sequences. We compared our tracker with several state-of-the-art trackers: SeqTrack [82], DropTrack [83], OSTrack [22], GRM [23], SwinTrack [33], CSWinTT [84], STARK [85], TransT [4], Ocean [73], SiamFC++ [74], DiMP-50 [18], and SiamDW [77]. Similar to the TrackingNet benchmark, the evaluation metrics include Precision and AUC scores. Detailed comparison results are presented in Table 4.

From the data in Table 4, it can be observed that our tracker achieves the highest score in terms of AUC scores, outperforming DropTrack and TransT by a margin of 0.1%. Although our tracker does not achieve the highest score in Precision, the difference from the top three trackers (Ocean, DropTrack, and SwinTrack) is relatively small. The lower performance in Precision may be attributed to the inability of domain adversarial training to eliminate overfitting of the model on synthetic data. Despite performing well on synthetic images during training, the model still struggles to accurately predict the position of the target in real images during testing. This observation is supported by the feature visualization experiment in Section 4.3.2, where some disparities still exist after domain adversarial training despite the significant reduction in differences between synthetic and real images. In short, the performance comparison data indicate that our tracker achieves competitive results on OTB100 compared to state-of-the-art trackers, demonstrating the effectiveness and feasibility of our approach.

**Figure 6:**
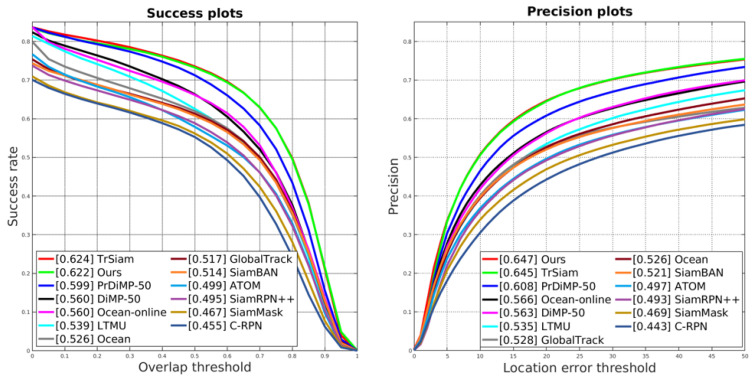
Plot showing the results on the LaSOT [64] testing set in terms of Precision and Success (AUC) scores.

**LaSOT** [64]. To verify that our tracker can maintain its excellent performance in long video tracking, we evaluated it on the test split of the LaSOT dataset. This split comprises 280 long videos with an average of 2512 frames per video. To compare our tracker with other top-performing trackers, we visualize the Success and Precision plots of each tracker in Figure 6. These trackers include TrSiam [79], PrDiMP-50 [72], DiMP-50 [18], Ocean [73], LTMU [86], and ATOM [78], among others.

From Figure 6, it is evident that our tracker demonstrates a strong competitive edge compared to others. In the Success plots, our tracker and TrSiam lead significantly ahead of other trackers, with our tracker trailing TrSiam by only 0.2%. Moreover, in the Precision plots, our tracker outperforms all other trackers, ranking first. This series of leading results convincingly demonstrates that our tracker maintains a robust competitive edge in long video tracking.

**UAV123**. The UAV123 dataset primarily comprises 91 drone videos, and is specifically designed to assess the robustness of a tracker against rapid changes in tracking perspectives. We evaluated our tracker on the UAV123 dataset and compared its performance with other top-performing trackers, including SiamTPN [69], TrTr [3], Ocean [73], and SiamBAN [87], among others. Detailed comparison results are presented in Table 5.

From the data in Table 5, it can be observed that while our tracker exhibits some weaknesses compared to the evaluations on the previous datasets, it remains highly competitive. In terms of Precision, our tracker shows relatively lower performance, which can be attributed to incomplete elimination of the impact of synthetic data during the adversarial training process. This may result in the feature extractor failing to effectively capture discriminative features within small targets, adversely affecting the prediction of target positions. However, our tracker still demonstrates excellent performance in terms of AUC score, ranking second among all compared trackers and with only a 0.7% difference from the top performer. These results effectively demonstrate the robustness of our tracker in handling rapid changes in tracking perspectives.

**Table 3:** Comparison results on the TrackingNet [65] dataset. The top three scores are highlighted in red, blue, and green, respectively, while * indicates that labeled data were not used for training.

Type	Supervised Method										Self-Supervised Method			
Trackers	Ours	BANDT [68]	TrTr [3]	TrDiMP [79]	KeepTrack [81]	PrDiMP-50 [72]	SiamFC++ [74]	DiMP-50 [18]	SiamRPN++ [75]	SiamDW [77]	Ours	ECO * [88]	LUDT * [89]	USOT * [9]
Precision (%)	74.9	74.5	67.4	73.1	73.8	70.4	68.7	70.5	69.4	56.3	60.4	48.9	49.5	55.1
Norm.prec (%)	83.5	82.7	79.5	83.3	83.5	81.6	80.0	80.1	80.0	71.3	73.2	62.1	63.3	68.2
Success (AUC) (%)	78.7	78.5	70.7	78.4	78.1	75.8	75.4	74.0	73.3	61.1	66.5	56.1	56.3	59.9

**Table 4:** Comparison results on the OTB100 [61] dataset. The top three scores are highlighted in red, blue, and green, respectively.

Trackers	Ours	SeqTrack [82]	DropTrack [83]	OSTrack [22]	GRM [23]	SwinTrack [33]	CSWinTT [84]	STARK [85]	TransT [4]	Ocean [73]	SiamFC++ [74]	DiMP-50 [18]	SiamDW [77]
Precision (%)	90.0	89.1	91.0	88.7	90.0	90.2	87.2	88.4	89.9	92.0	89.6	88.8	89.2
Success (AUC) (%)	69.6	68.3	69.5	68.1	68.9	69.1	67.1	68.0	69.5	68.4	68.3	68.0	67.0

**Table 5:** Comparison results on the UAV123 [62] dataset. The top three scores are highlighted in red, blue, and green, respectively.

Trackers	Ours	SiamTPN [69]	TrTr [3]	Ocean [73]	SiamFC++ [74]	SiamBAN [87]	DiMP-50 [18]	SiamDW [77]	HiFT [80]
Precision (%)	82.7	82.3	83.9	82.3	81.0	83.3	85.8	77.6	78.7
Success(AUC) (%)	64.1	63.6	63.3	62.1	62.3	63.1	64.8	53.6	58.9

### 4.5. Qualitative Comparison

**Figure 7:**
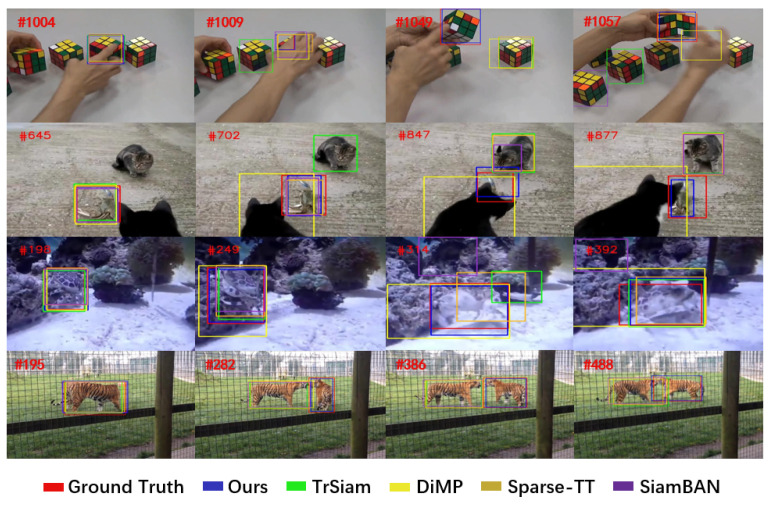
Qualitative comparison results between our tracker and four other representative trackers on the LaSOT benchmark.

Finally, to demonstrate the effectiveness of our tracker in complex scenarios, we visualize the tracking results of our tracker and four other representative trackers on the LaSOT dataset. Detailed results are shown in Figure 7. From the visualizations, in the first and fourth rows it can be seen that our tracker exhibits better robustness when facing interference from similar objects. In addition to interference from similar objects, target deformations and occlusions are common occurrences in real-world scenarios. From the results in the second and third rows, it is evident that our tracker continues to accurately track the target, demonstrating strong robustness even when the tracked target undergoes significant deformation or is occluded by other objects. These visualization results indicate that our tracker maintains strong robustness in various complex scenarios.

## 5. Limitations, Discussion, and Future Work

Although our method successfully eliminates the need for manual annotations and mitigates domain discrepancies between synthetic and real images through domain adversarial learning, a significant performance gap remains compared to fully supervised trackers. This limitation arises from two main factors. First, while synthetic training data can effectively provide complete target coverage, they may not fully capture the complex dynamics, appearance variations, and background clutter present in real-world videos, limiting the model’s generalization ability. Second, domain adversarial training, although beneficial, cannot entirely remove subtle distribution differences, and in some cases may lead to unstable convergence or reduced feature discriminability. As a result, the learned representations may fall short in challenging tracking scenarios involving occlusion, motion blur, or similar distractors.

Essentially, training a visual object tracker requires knowledge of the target’s location in video frames, including both the template and the search frames. This requirement fundamentally conflicts with the definition of self-supervised learning, which assumes no access to labeled data. Therefore, we argue that self-supervised training for visual tracking should be regarded as a form of pretraining rather than as a standalone training paradigm. This perspective aligns with recent self-supervised approaches such as MAE [90], where the goal is to learn transferable representations that can benefit downstream tasks.

In our future work, we plan to introduce more advanced image synthesis techniques such as conditional diffusion models; specifically, these techniques allow the diffusion model to generate more realistic images by conditioning the generation process on the target template and its spatial location, then treating the video frames to be synthesized as noisy inputs. This is expected to further narrow the gap between synthetic and real data, thereby enhancing the generalization ability of self-supervised training. Additionally, we plan to adopt more powerful domain adaptation strategies and explore multimodal self-supervised learning approaches, with the goal of extending the applicability of self-supervised training to a broader range of real-world scenarios.

## 6. Conclusions

With the continuous growth of applications based on sensors, the demand for visual tracking has become increasingly urgent. This paper explores methods for training an efficient deep visual tracking model in the absence of a large amount of labeled data. To this end, we propose a self-supervised method based on image synthesis and domain adversarial training. The proposed method aims to conduct self-supervised training using complete targets while also achieving effects on deep networks. Our method first builds a diverse database of target objects and synthesizes training pairs by embedding complete targets into unlabeled video frames, thereby ensuring precise bounding box supervision. Then, we incorporate domain adversarial training into the model optimization process to narrow the domain gap between synthetic data and real data. Experimental results show that our method achieves an AUC score of 66.5% on the TrackingNet dataset without using the labeled data, showing competitive performance compared to supervised benchmarks, while significantly reducing the gap between self-supervised tracking and supervised tracking. The proposed method can provide an efficient solution for AI-driven visual tasks where labeled data are scarce and unlabeled data remain underexploited.

## Figures and Tables

**Table 1:** Ablation study results on the OTB100 dataset [61]. Checkmarks indicate that the module is used, and the highest score is indicated in bold.

Domain Adversarial	Image Transformation	Precision (%)	AUC (%)
		86.2	65.1
✔		88.1	67.3
	✔	88.6	67.2
✔	✔	**90.0**	**69.6**

**Table 2:** Comparison results on the GOT10k [63] test set. The top three scores are highlighted in red, blue, and green, respectively.

Trackers	AO (%)	SR_0.50_ (%)	SR_0.75_ (%)
BANDT [68]	64.5	73.8	54.2
SiamTPN [69]	57.6	68.6	44.1
SiamGAT [70]	62.7	74.3	48.8
SiamR-CNN [71]	64.9	72.8	59.7
PrDiMP-50 [72]	63.4	73.8	54.3
Ocean [73]	61.1	72.1	47.3
SiamFC++ [74]	59.5	69.5	47.3
DiMP-50 [18]	61.1	71.7	49.2
SiamDW [77]	42.9	48.3	14.7
ATOM [78]	55.6	63.4	40.2
SiamRPN++ [75]	51.7	61.6	32.5
STMTrack [76]	64.2	73.7	57.5
Ours	66.4	77.9	59.2

## Data Availability

The LaSOT dataset is available at http://vision.cs.stonybrook.edu/~lasot/, the TrackingNet dataset is available at https://eval.ai/web/challenges/challenge-page/1805/overview, the OTB100 dataset is available at https://github.com/prosti221/OTB-dataset, and UAV123 dataset is available at https://irip-buaa.github.io/posts/UAV123/.
